# Development and application of an automated, multiwell plate based screening system for suspension cell culture

**DOI:** 10.1186/1753-6561-7-S6-P113

**Published:** 2013-12-04

**Authors:** Sven Markert, Carsten Musmann, Klaus Joeris

**Affiliations:** 1 Roche Diagnostics GmbH, Pharma Biotech Production and Development, Penzberg, Germany

## Introduction

The already presented automated, multiwell plate (MWP) based screening system for suspension cell culture is now routinely used in process development. It is characterized by a fully automated workflow with integrated analytical instrumentation and uses shaken 6-24 well plates as bioreactors which can be run in batch and fed-batch mode with a capacity of up to 384 reactors in parallel [1].

A wide ranging analytical portfolio is available to monitor cell culture processes and to characterize product quality. Assays running on the screening system comprise the determination of cell concentration and viability, quantification of nutrients and metabolites as well as detection of apoptosis level and staining of organelles. Additionally a RT-qPCR method has been setup to measure gene expression level in a high throughput manner. Having a large network in-house to high throughput groups of the analytical department a lot of advanced methods can easily be performed like chromatographic and mass spectrometry to characterize product quality.

Current work focuses on expanding the analytical portfolio to develop control strategies for automated cell culture processes. Besides setting up a robust method for pH measurement we evaluate different spectroscopic techniques like Raman, infrared or 2D fluorescence as fast and powerful analytical tools.

## Results

### Scale-up prediction

The comparability of results obtained with multiwell plates and bioreactors had to be verified to develop a screening system for the predictive scale-up.

Using several late stage project cell lines growing in suspension the comparability of results obtained with automated, shaken multiwell plates and bioreactors with a volume of up to 1.000 L could be verified. The effects of process optimization steps on cell culture performance and product quality were shown in multiwell plates and bioreactors. Thus, the automated cell culture screening system can be used for scale up prediction.

### Application of pH measurement and pH control

A fully automated, multiwell plate based pH measurement assay and a pH control strategy was developed for the screening system. The established assay is based on the use of pH sensitive absorption and fluorescent dyes which are added to a cell culture sample. The advantages of this method comprise a short analytical time and the low sample volume per sample. The assay is characterized by a high precision and robustness without any probe drift during a cultivation time of up to two weeks.

**Figure 1:**
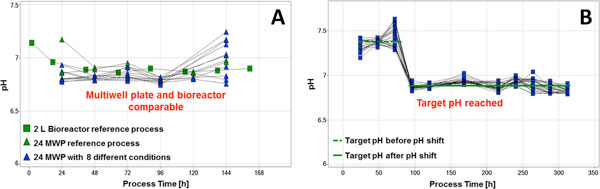
**(A) Comparability of the pH profile between the MWP reference process and the 2L bioreactor reference process**. Additionally further pH profile and product concentration under different media compositions. **(B) **pH sensitive process with pH shift. The target pH, before and after the shift, was achieved by pH control.

The successful application of the developed pH measurement and pH control could be confirmed by getting comparable pH profiles from MWP and bioreactor under the same conditions and can be kept equal by controlling the pH (Figure 1A).

In a second experiment a pH shift of 0.4 pH values after 72 hours was performed (Figure 1B). The target pH was reached exactly and it could be controlled at a stable level using the developed pH measurement assay.

### Feasibility of Raman spectroscopy as high throughput analytical tool

Raman spectroscopy is a powerful tool for the detection and quantification of several components in cell culture processes at once. Using this fast and non-invasive analytical technique there will be no reagent costs and no sample consumption what this technique makes ideal for small scale high throughput systems.

The feasibility of Raman spectroscopy was shown for the quantification of different metabolites and nutrients, i.e. glucose, lactate and glutamine. For the quantification of glucose (0 g/L to 20g/L), lactate (0 g/L to 10 g/L) and glutamine (0 g/L to 20 g/L) a good correlation with a high prediction accuracy could be shown.

## Conclusions and outlook

The developed robotic screening system is capable of performing a fully automated workflow consisting of incubation, sampling, feeding and near real-time analytics. In the performed experiments the scalability from mL scale up to 1000 L scale could be shown.

Expanding the analytical portfolio a robust and fast pH measurement assay was developed to enable pH control in multiwell plates. This assay as well as pH control was tested during the cultivation of two late stage project cell lines resulting in comparable pH profiles and cell culture performance. These results enable the routinely use of the developed pH measurement and control strategy. Additionally the proof of concept for Raman spectroscopy as a powerful tool for the quantification of metabolites and nutrients for the automated screening system could be shown. Further spectroscopic techniques using infrared or fluorescence will be evaluated.
